# Sentiment in nursing notes as an indicator of out-of-hospital mortality in intensive care patients

**DOI:** 10.1371/journal.pone.0198687

**Published:** 2018-06-07

**Authors:** Ian E. R. Waudby-Smith, Nam Tran, Joel A. Dubin, Joon Lee

**Affiliations:** 1 Department of Statistics and Actuarial Science, University of Waterloo, Waterloo, Ontario, Canada; 2 Health Data Science Lab, School of Public Health and Health Systems, University of Waterloo, Waterloo, Ontario, Canada; University Antwerp, BELGIUM

## Abstract

**Background:**

Nursing notes have not been widely used in prediction models for clinical outcomes, despite containing rich information. Advances in natural language processing have made it possible to extract information from large scale unstructured data like nursing notes. This study extracted the sentiment—impressions and attitudes—of nurses, and examined how sentiment relates to 30-day mortality and survival.

**Methods:**

This study applied a sentiment analysis algorithm to nursing notes extracted from MIMIC-III, a public intensive care unit (ICU) database. A multiple logistic regression model was fitted to the data to correlate measured sentiment with 30-day mortality while controlling for gender, type of ICU, and SAPS-II score. The association between measured sentiment and 30-day mortality was further examined in assessing the predictive performance of sentiment score as a feature in a classifier, and in a survival analysis for different levels of measured sentiment.

**Results:**

Nursing notes from 27,477 ICU patients, with an overall 30-day mortality of 11.02%, were extracted. In the presence of known predictors of 30-day mortality, mean sentiment polarity was a highly significant predictor in a multiple logistic regression model (Adjusted OR = 0.4626, p < 0.001, 95% CI: [0.4244, 0.5041]) and led to improved predictive accuracy (AUROC = 0.8189 versus 0.8092, 95% BCI of difference: [0.0070, 0.0126]). The Kaplan Meier survival curves showed that mean sentiment polarity quartiles are positively correlated with patient survival (log-rank test: p < 0.001).

**Conclusions:**

This study showed that quantitative measures of unstructured clinical notes, such as sentiment of clinicians, correlate with 30-day mortality and survival, thus can also serve as a predictor of patient outcomes in the ICU. Therefore, further research is warranted to study and make use of the wealth of data that clinical notes have to offer.

## Introduction

Common methods of predicting mortality in the intensive care unit (ICU) often use severity of illness scores (SOI), such as APACHE [1], SAPS [2] or SOFA [3]. These SOI systems are based on coded data of the patient’s demographics, lab test results and vital signs that are now commonly available from a patient’s electronic health record (EHR). However, common EHRs also contain unstructured data (not in any organized forms such as a table) like clinical notes written by clinicians that are often not used in mortality prediction. Previous studies suggest that clinicians are reasonably capable of predicting mortality in the ICU [4, 5], therefore their notes should also offer valuable information about the patient’s health status.

Previous studies found that the sentiments—impressions or attitudes—of the clinicians toward individual patients can be measured via *sentiment analysis*, a method to quantify or categorize subjective properties of written text [6, 7]. Sentiment is often expressed as the relative polarity or positivity of a string of text as a number from -1 (very negative) to 1 (very positive) [7]. Alternatively, sentiment may be interpreted as the estimated probability of being classified as “positive” rather than “negative” by some classifier. Sentiments measured in clinical notes were found to differ by clinical outcomes, demographic features, and over time [6], and be associated with readmission [8] and mortality [9] [8]. In health applications, sentiment analysis has been used to study the relationship between happiness and geographical factors using Twitter [10], sentiment of cancer survivors online [11, 12], the patient experience from comments online [13], health care reform on Twitter [14], smoking behavior and perceptions of tobacco products on Twitter [15], tweets about pandemics [16], and sentiments about vaccinations [17].

This study explores the association between the sentiment measured in clinical notes and mortality risk by considering nursing notes obtained from MIMIC-III, an open database that contains ICU patient data from the Beth Israel Deaconess Medical Center between 2001—2012 [18]. We hypothesize that sentiment measured in nursing notes can also offer a high degree of predictive power for mortality risk because: nurses have more consistent direct contact with patients than any other clinical professionals [19]; nurses frequently detect signs of health deterioration in patients [19, 20]; and nurses can highlight any abnormal clinical measures and record their judgments or concerns in their documentation [21].

This study applied a sentiment analysis algorithm to a corpus of nursing notes and correlated the sentiment with 30-day mortality (expired versus survived) while controlling for gender, type of ICU, and SAPS-II score. In addition, statistical hypothesis tests were performed to examine whether patients that survived or expired 30 days post-admission had significant differences in mean sentiment. The association between sentiment and 30-day mortality was further examined in assessing the contribution toward predictive performance of sentiment score as a feature in a classifier, and in a survival analysis for different levels of sentiment.

## Materials and methods

### Data extraction

Patient and nursing note data were extracted from MIMIC-III, an openly available database developed by the MIT Lab for Computational Physiology [18]. The database contains rich de-identified health data associated with more than 40,000 patients who were admitted to the ICUs of the Beth Israel Deaconess Medical Center between 2001 and 2012 [18]. MIMIC-III data contains demographics, vital signs, laboratory tests, transcribed nursing notes (usually available for each 8-hour shift for patients), and mortality (in and out-of hospital) of patients admitted into the ICUs [18].

It is important to note that dates of birth for MIMIC-III patients older than 89 were shifted to keep patients anonymous and comply with the Health Insurance Portability and Accountability Act [22]. Therefore, we assigned a date of birth to those patients by random sampling with replacement following the empirical age distribution of patients older than 89 in MIMIC-II (the previous version of the MIMIC-III database, where dates of birth were available for all patients aged 89 or older that had already died) for male and female groups separately [23]. However, this only affects our reported age demographics as age was not included in our models.

MIMIC-III (and MIMIC-II) are open datasets in the public domain, therefore research ethics review was not needed for this study. Data were extracted from the MIMIC databases using Structured Query Language (SQL) in pgAdmin III (version 1.22.1).

### Inclusion/Exclusion criteria

Since the interested outcome is mortality post-admission, this study only considered the nursing notes from the first hospital admissions to eliminate duplicated observations from patients that may have multiple admissions.

This study only considered adult patients (aged 15 or older at hospital admission). The patients that were admitted to the coronary care unit (CCU), cardiac surgery recovery unit (CSRU), medical ICU (MICU), surgical ICU (SICU), or trauma/surgical ICU (TSICU) were included in this study. In the MIMIC-III database, patients admitted between 2001 and 2008 were followed for a maximum of 4 years for out-of-hospital mortality, while those admitted after 2008 had a shorter maximum follow-up of 90 days [22]. This difference in maximum follow-up time is a result of patient data being stored in two different clinical information systems based on when they were admitted to the ICU [22]. Patients from both admission periods were included in this study.

Notes identified by physicians as errors (indicated by the *iserror* column in the MIMIC-III nursing notes table [22]) were excluded. Notes that were written fewer than 12 hours prior to the time of death were also excluded to reduce a potential skewness in the sentiment. In addition, this study excluded patients without any nursing notes data.

### Sentiment analysis

There are two primary techniques for classifying and computing sentiment polarity in text: semantic and syntactic [24]. The semantic approach aims to extract sentiment based on the meaning of the text, and is most commonly achieved via using a classifier that was trained on a set of labelled and annotated text [24]. Since there is not a pre-labelled/annotated set of nursing notes available to train a classifier, this study focused on the syntactic approach, which aims to make inferences based on the structural features of the text [24, 25].

Sentiment scores for the nursing notes were computed using the Python programming language (version 3.6.4) and the TextBlob natural language processing library (version 0.15.1) [26]. To employ a syntactic approach to sentiment analysis, TextBlob uses the Pattern module to compute the sentiment of strings of text [25]. Pattern contains a lexicon for many English-language adjectives and adverbs, each of which is mapped to “polarity”, “subjectivity”, and “intensity” scores [27]. Given a string of text, Pattern uses a part-of-speech tagger to identify adjectives and adverbs whose polarity, subjectivity, and intensity scores are retrievable from the lexicon [28]. Polarity and subjectivity of adjectives may be adjusted according to the intensity of modifying adverbs (multiplies by a factor between 1/2 and 2), and/or the presence of negations (reverses polarity) [28] [27]. Pattern then computes polarity and subjectivity scores for the entire string as an average of the (potentially modified) scores for adjectives therein [28]. TextBlob returns sentiment polarity as a score from -1 to 1 and sentiment subjectivity as a score from 0 to 1 with higher scores representing more positive, and more subjective sentiment, respectively. For specific details on the algorithm’s implementation or the English-language lexicon used, we direct the reader to the official git repository for Pattern.

In this study, each individual nursing note was assigned a sentiment polarity score and a sentiment subjectivity score. Scores were computed by creating a TextBlob object initialized with a nursing note string and extracting the ‘sentiment’ attribute from said object. Prior to initializing TextBlob objects, no pre-processing was performed on the notes. For every patient’s first hospital admission, the mean sentiment polarity (and subjectivity) of notes taken during that admission was calculated. Mean sentiment polarity and mean sentiment subjectivity scores were then used as predictors in our models. To compare average sentiment polarity / subjectivity between the survived and expired groups, we performed two-tailed, unpaired, unequal variance *t*-tests.

**Fig 1:**
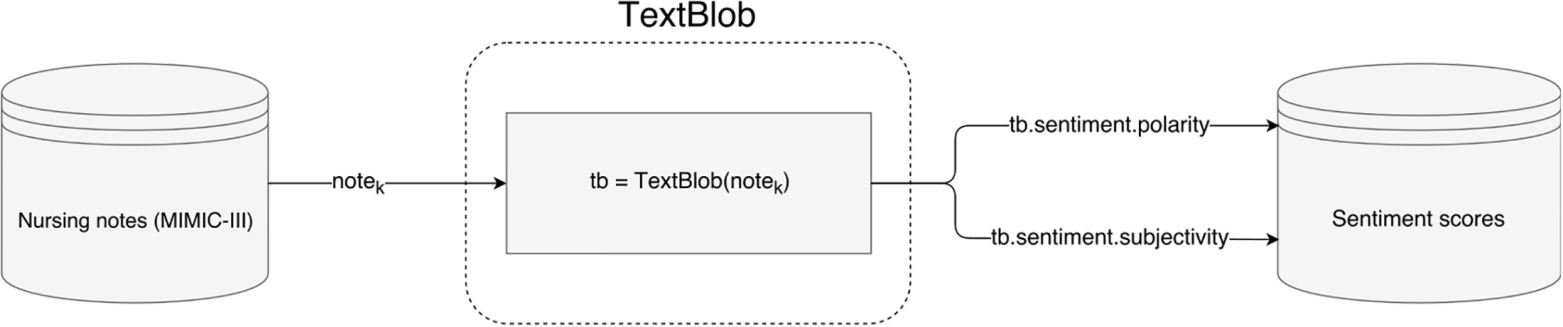
Flowchart illustrating the process of computing sentiment scores of nursing notes using the TextBlob library.

An illustration of the process used in this study to compute and store sentiment of notes can be seen in Fig 1. Example excerpts from MIMIC-III nursing notes and their computed sentiment scores are shown in Table 1.

**Table 1:** Sample excerpts from ICU nursing notes in MIMIC-III, and their corresponding sentiment polarity scores. Adjectives and Adverbs Contributing to Sentiment are in Bold with a 2-tuple of Polarity and Subjectivity Scores.

Excerpt Text with De-identified Patient Information	Sentiment Polarity	Sentiment Subjectivity
“Pt is alert and oriented, **pleasant**^(0.733, 0.967)^, worried about being in the ICU but is comforted by her family. CV: Her SBP has been 70s-90s,per a note from the [**Hospital1 **] she often runs lower that the 90s for a sytolic and her BP on that office visit was in the 70s. Her HR has been 80s-90s, she is mentating well.”	0.7333	0.9667
“Extremities mottled. Family **aware**^(0.25, 0.25)^ of **grim**^(−1.0, 1.0)^ condition-they requested he receive comfort measures **only**^(0.0, 1.0)^. MSO4 drip begun and pressors off. Family at bedside.”	-0.25	0.75
“Reason for admission: Mr. [**Known lastname 10770**] is a 25 year **old**^(0.1, 0.2)^ man admitted to [**Hospital1 1**] on [**2126-2-1**] with 7 gun shot wounds to his torso, R elbow and R wrist. Injuries include: duodenal injury, renal vein laceration, R elbow fx, R wrist fx”	0.1	0.2

### Mortality prediction and survival analysis

We fit a multiple logistic regression model to our data and computed adjusted odds ratios with 30-day mortality (‘expired’ and ‘survived’ coded as 1 and 0, respectively) as the response variable and the following features: mean sentiment polarity score (-1 to 1), mean sentiment subjectivity score (0 to 1), SAPS-II (0 to 163, least to most severe) [2], gender (categorical), and first ICU type (categorical) after hospital admission. This study considered a significance level at p ≤ 0.05.

Since a one-unit increase in mean sentiment polarity score is half the range (and in the case of subjectivity, the entire range) of possible values, the adjusted odds ratios for these features may be very small and difficult to interpret. Therefore, we scaled mean sentiment polarity and subjectivity scores by a factor of 10 to enhance interpretability and correspond to 0.1-unit increases in mean sentiment polarity and subjectivity, respectively.

SAPS-II is a known predictor of ICU mortality and a composite score which accounted for the patient’s age, 12 physiological variables, type of admission, and 3 co-morbidity variables [2]. The SAPS-II score was calculated using data available in MIMIC-III and SQL scripts found in the MIT Laboratory for Computational Physiology Git repository. Gender and ICU type were variables available from MIMIC-III but not included in SAPS-II, therefore this study included these to control for these additional factors.

To further assess the predictive power of sentiment polarity scores, this study trained a multiple logistic regression classifier with sentiment scores as features and one without, both with 50 replications of 10-fold cross validation. The other features that were included are SAPS-II scores, gender, and ICU type. The benchmark performance measures were the areas under the Receiver Operating Characteristic Curve (AUROC) and Precision Recall Curve (AUPRC), respectively. We then computed a 95% bootstrap confidence interval (BCI) for the difference in AUROC (AUROC_diff_ = AUROC_(sentiment)_ − AUROC_(w/o sentiment)_) and for the difference in AUPRC (AUPRC_diff_ = AUPRC_(sentiment)_ − AUPRC_(w/o sentiment)_) using 2000 resamples.

In addition, this study partitioned patients based on their mean sentiment polarity and subjectivity quartiles, and then compared the survival curves from the quartiles using the Kaplan-Meier curve [29] and a log-rank test. Survival was calculated as the number of days between hospital admission and the time of death or right-censoring. Since patients had different follow-up times for out-of-hospital mortality based on their date of admission, survival was right-censored appropriately. Specifically, patients admitted between 2001 and 2008, and after 2008 were right-censored at 4 years, and 90 days, respectively for the survival analysis in this study.

## Results

This study included 27,477 patients from the selected ICUs that satisfied all the inclusion/exclusion criteria. The overall 30-day mortality of this cohort is 11.02%. The Spearman rho rank correlation between mean sentiment polarity and SAPS-II was found to be -0.2251 (p < 0.001), while that between mean sentiment subjectivity and SAPS-II was found to be 0.0138 (p = 0.022).

**Fig 2:**
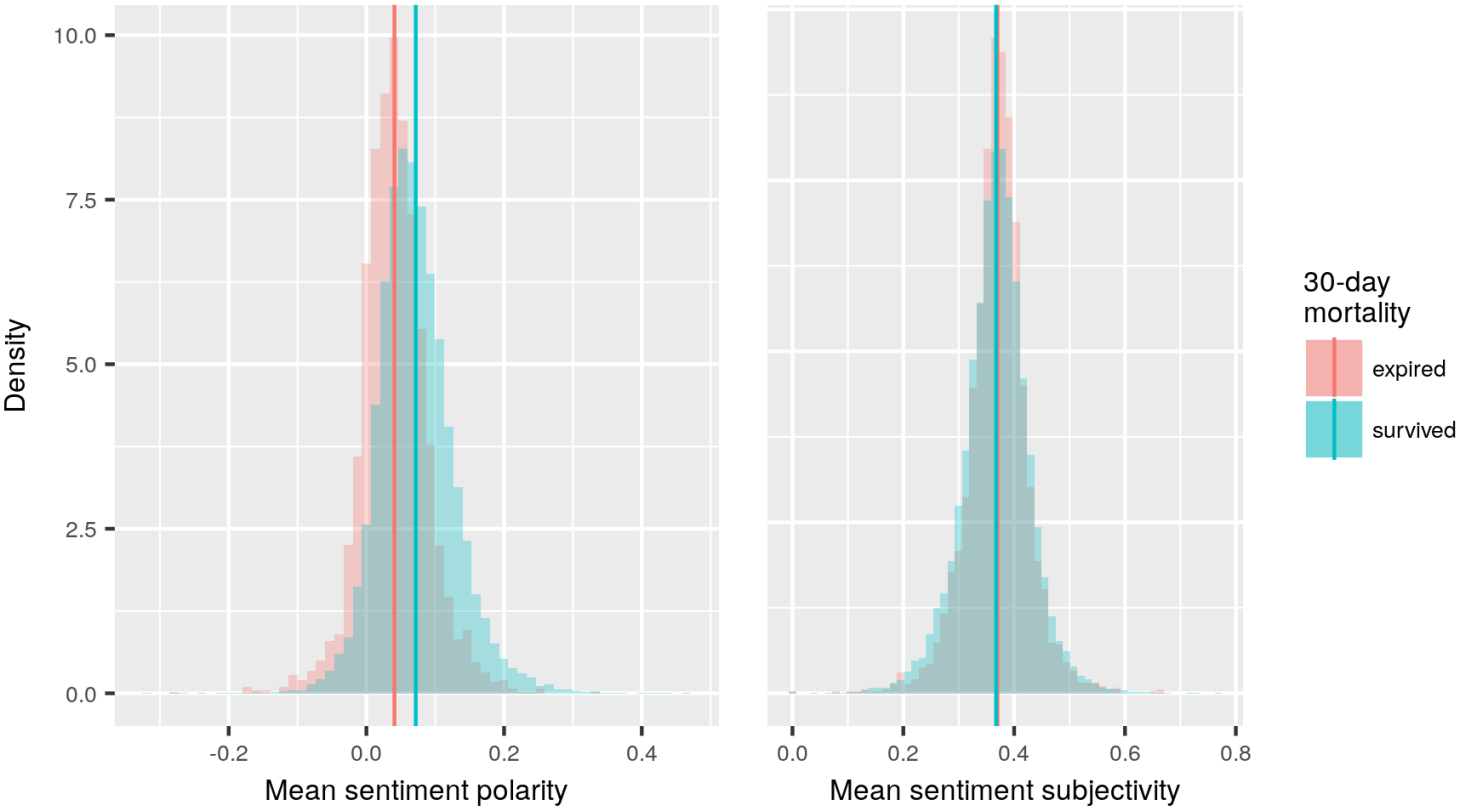
Density histograms of mean sentiment polarity (Left) and subjectivity (Right) scores for patients that expired within 30 days and patients that survived.

Nursing notes of patients that survived the 30-day post-admission period had higher mean sentiment polarity scores than notes of patients that expired (means: 0.0717 and 0.0407 respectively; *t*-test: p < 0.001). In contrast, the survived group had lower mean sentiment subjectivity scores than the expired group (means: 0.3674 and 0.3700, respectively; *t*-test: p = 0.0172) (Fig 2). Additionally, a density histogram showed that the mean sentiment polarity of both the survived and expired groups share similar-shaped (seemingly Gaussian) distributions, with mean sentiment polarity score in the survived group higher than the expired group (Fig 2). The distribution of mean sentiment subjectivity scores in the survived and expired groups also have seemly Gaussian distributions but with much closer means.

Summary statistics of clinical and demographic variables from the cohort are presented in Table 2. In addition, p-values obtained from hypothesis tests for comparing these variable values between 30-day mortality groups are provided. Numerical variables are compared using two-tailed unpaired, unequal variance *t*-tests, while categorical variables are compared using Pearson’s (asymptotic) *χ*^2^ test of independence.

**Table 2:** Summary statistics of clinical and demographic variables for eligible patients, broken down by 30-day mortality group with p-values from hypothesis tests for comparing variable values between groups.

	Survived	Expired	p-value
Number of patients	24448	3029	-
Mean sentiment polarity (mean [standard deviation])	0.072	0.041	< 0.001
Mean sentiment subjectivity (mean [standard deviation])	0.367	0.370	0.017
ICU type			< 0.001
CCU (%)	15.69	15.55	
CSRU (%)	22.95	6.80	
MICU (%)	32.34	48.93	
SICU (%)	15.07	17.33	
TSICU (%)	13.96	11.39	
SAPS-II (mean [standard deviation])	32.02 [12.82]	46.94 [14.37]	< 0.001
Age (mean [standard deviation])	62.47 [17.80]	71.85 [15.75]	< 0.001
Gender (Female) (%)	42.48	46.72	< 0.001
Number of nursing notes during first admission (mean [standard deviation])	16.68 [28.91]	20.85 [23.71]	< 0.001

Adjusted odds ratios for the multiple logistic regression model fit are displayed in Table 3. For this model output, mean sentiment polarity and subjectivity were scaled by a factor of 10 to improve the interpretability of odds ratio estimates. The multiple logistic regression model fit shows that mean sentiment polarity score is inversely associated with 30-day mortality while higher subjectivity is positively associated with 30-day mortality (Table 3). As expected, SAPS-II scores were found to be positively associated with 30-day mortality. Gender was not found to be statistically associated with 30-day mortality in these models. Patients that stayed at the CSRU were least likely to expire during the 30-day post-admission period, while patients from the MICU were most likely to expire within this period (Table 3).

**Table 3:** Adjusted odds ratios of the features in a fitted multiple logistic regression model and their corresponding 95% confidence intervals and p-values.

	Odds Ratio	2.5%	97.5%	p-value
Scaled Mean Sentiment Polarity	0.4626	0.4244	0.5041	< 0.001
Scaled Mean Sentiment Subjectivity	1.2016	1.1150	1.2952	< 0.001
SAPS-II	1.0712	1.0679	1.0744	< 0.001
Gender				
Female	Referent	-	-	-
Male	1.0006	0.9204	1.0880	0.9879
ICU Type				
CCU	Referent	-	-	-
CSRU	0.2997	0.2502	0.3580	< 0.001
MICU	1.3167	1.1671	1.4878	< 0.001
SICU	1.2674	1.0973	1.4645	0.0013
TSICU	1.1171	0.9530	1.3086	0.1709
Deviance	15293 on 27468 degrees of freedom			>0.999
Nagelkerke Pseudo-*R*^2^	0.2566			

The multiple logistic regression classifier that was trained with sentiment scores (mean sentiment polarity and subjectivity) as features shows improvements in AUROC and AUPRC over the classifier trained without (AUROC_diff_ 95% BCI: [0.0070, 0.0126], AUPRC_diff_ 95% BCI: [0.0159, 0.0261]) (Table 4). The relatively small standard deviations in AUROC and AUPRC indicate that both classifiers were relatively stable in predicting mortality (Table 4).

**Table 4:** Areas Under the Receiver Operating Characteristic Curve (AUROC), and Precision Recall Curve (AUPRC) in 10-fold cross validation with 50 replications of multiple logistic regression classifiers.

Features	AUROC (stdev)	AUPRC (stdev)
SAPS-II, Gender, ICU Type, Mean Sentiment Polarity, Mean Sentiment Subjectivity	0.8189 (0.0106)	0.3698 (0.0243)
SAPS-II, Gender, ICU Type	0.8092 (0.0117)	0.3495 (0.0236)
Difference	0.0097, 95% BCI: [0.0070, 0.0126]	0.0203, 95% BCI: [0.0159, 0.0261]

stdev, standard deviation

**Fig 3:**
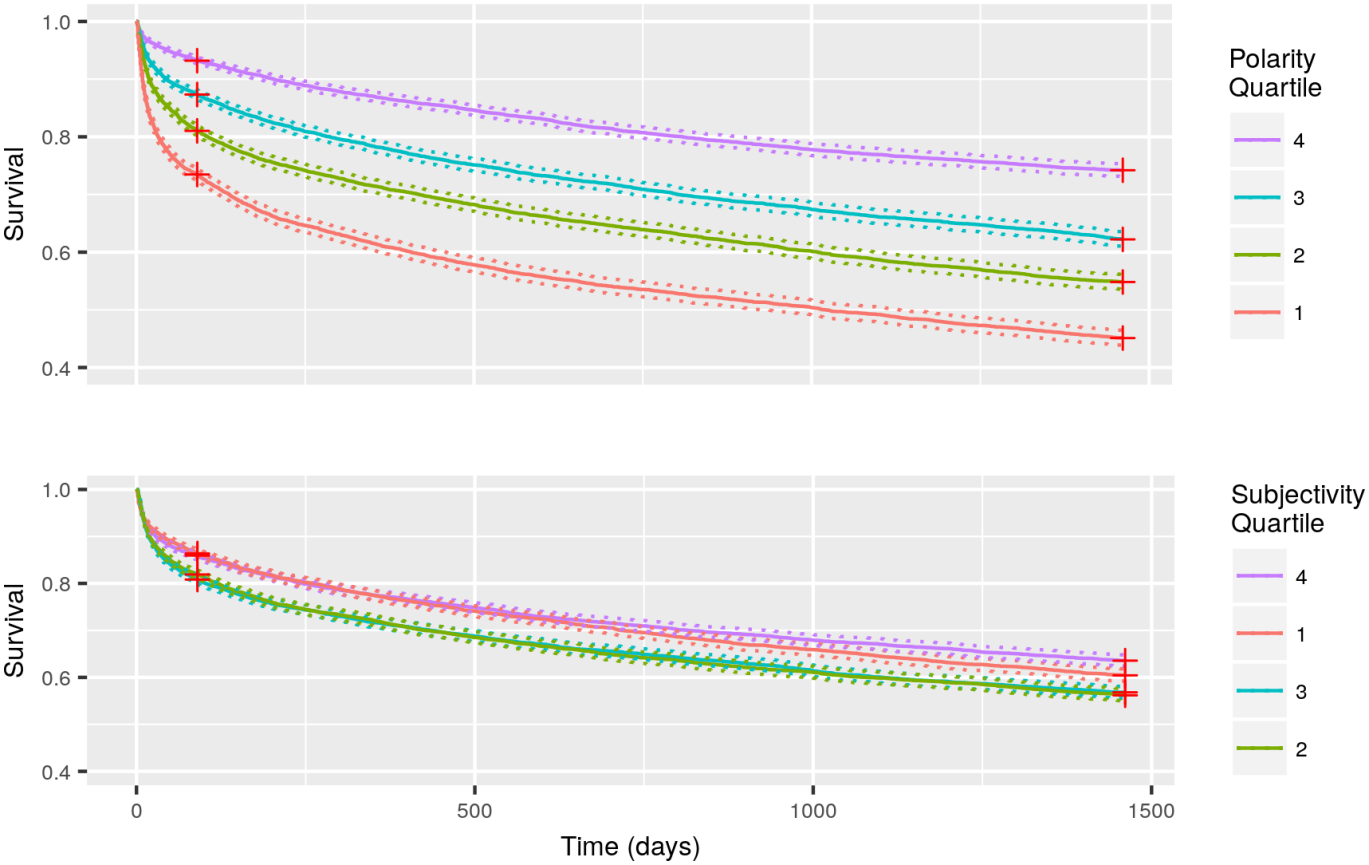
Kaplan Meier survival curves for patients partitioned by their mean sentiment polarity (Top) and subjectivity (Bottom) quartiles. Red Crosses Represent Patients That Were Right-censored at 90 Days and 4 Years Post-Admission, and Dash Lines Represent 95% Confidence Intervals.

When patients were partitioned into their respective mean sentiment polarity quartiles, the Kaplan-Meier survival curves showed clear separations among the quartile groups (log-rank test: p < 0.001) (Fig 3). Specifically, the first quartile showed the worst survival, and the fourth quartile showed the highest survival, with the second and third quartiles falling expectedly and proportionally in the middle (Fig 3). On the other hand, partitioning patients by mean sentiment subjectivity quartiles did not yield clear or proportional separations among all four groups. However, the first and fourth quartiles appear to have slightly higher survival than the second and third (log-rank test: p < 0.001) (Fig 3), though we are uncertain of the reason for this specific pattern of mean sentiment subjectivity quartiles and survival experience.

## Discussion

The results show that even in the presence of known predictors of 30-day mortality, sentiment scores measured in nursing notes are statistically significant predictors, supported by the multiple logistic regression model (Table 3), and lead to improved mortality predictions as measured by AUROC and AUPRC (Table 4). Additionally, the results show that survival is positively correlated to mean sentiment polarity quartiles (Fig 3) but this relationship is much more limited in the case of mean sentiment subjectivity quartiles. These associations are consistent with a previous finding by McCoy et al. that found sentiment measured in discharge notes is associated with readmissions and mortality risk [8]. Taken together, the results suggest that clinical notes authored by hospital staff are informative and can serve as indicators of clinical outcomes, in addition to the information contained in structured clinical data such as vital signs, lab test results, diagnosis, etc.

By measuring clinicians’ sentiment, we are not just relying on their emotions and attitudes toward patients, but also their intuition and experience to predict outcomes for patients [19, 21], which were found to be reasonably accurate in previous research [4, 5, 30, 31]. Therefore, unstructured clinical notes should be further studied and incorporated in clinical outcomes prediction models, which have traditionally only used structured variables from EHRs.

There are also some limitations and important technical considerations. First, our technique used to compute sentiment is not the only technique available. Other techniques, such as those based on machine learning models to make semantic inferences, could yield different results.

Second, although sentiment scores improve the performance of our classifier as measured by *statistical* significance, it is not necessarily the case that all medical practitioners would deem this improvement *clinically* significant. Ultimately, the clinical significance of these results is to be determined by the medical expert and heavily depends on problem they wish to solve.

Third, the aggregated mean sentiment scores were only able to characterize the variation in sentiment at the patient-level, but not at the clinician-level, which would require clinician demographic data to be further studied. The variation in sentiment can also be found within a document at the sentence or paragraph-level. Therefore, due to being aggregated, the mean sentiment scores computed do not spread throughout all of the possible ranges (-1 to 1, or 0 to 1), and appear to be skewed toward the positive range in the case of sentiment polarity (Fig 2). Additionally, as a result of aggregation, subtle differences over time or in changes in health conditions may not have been captured by our study. Previous studies found that the patterns of nursing documentation could also be useful in predicting mortality [21, 32]. Therefore, different approaches in examining sentiment of clinical notes or other features, such as the frequency or contextual information, of the clinical notes can yield further insights.

Finally, despite the fact that MIMIC-III data are from a large-volume hospital with a considerable number of patients, the authors of the nursing notes in this study represent only a small cohort of nurses. Other cohorts of clinicians, with different training, experience, and working environment, may produce nursing notes with varied characteristics. The MIMIC-III database is from a single center, which may have an unique clinical culture that influenced how the nurses constructed the notes as well. Therefore, generalizability of the association between measured sentiment and mortality for nursing notes found elsewhere may be limited.

In spite of these limitations, the results demonstrated in the manuscript provide exciting potential for the inclusion of nursing notes in predictive models of patient mortality in the ICU.

Although the results are promising, the most important next step is to translate the knowledge gained into a practical solution to help clinicians make better decisions in the ICU. Some possible future steps include: automated predictive models that identify high-risk patients so that resources can be diverted to prevent adverse outcomes—as also suggested by McCoy et al. [8], and incorporating measured sentiment as a routinely monitored variable to serve as an indicator of health conditions for ICU patients. Additionally, it would be invaluable to further studies of sentiment analysis in medical notes if nurses were to assign their notes a “negative”, “positive”, or “neutral” score, effectively producing a labeled corpus of nursing notes.

Overall, although prediction of mortality and other clinical outcomes remains a complex problem, further improvements to predictive performance is possible by incorporating more information extracted from unstructured data, like nursing notes.
